# Genetic Characterization of the Central Variable Region in African Swine Fever Virus Isolates in the Russian Federation from 2013 to 2017

**DOI:** 10.3390/pathogens11080919

**Published:** 2022-08-15

**Authors:** Ali Mazloum, Antoinette Van Schalkwyk, Roman Chernyshev, Andrey Shotin, Fedor I. Korennoy, Alexey Igolkin, Alexander Sprygin

**Affiliations:** 1 Reference Laboratory for African Swine Fever Virus, FGBI “Federal Centre for Animal Health” (FGBI “ARRIAH”), 600901 Vladimir, Russia; 2 Agricultural Research Council-Onderstepoort Veterinary Institute, 100 Old Soutpan Road, Onderstepoort 0110, South Africa; 3 Department of Biotechnology, University of the Western Cape, Robert Sobukwe Road, Bellville 7535, South Africa

**Keywords:** African swine fever virus, central variable region, single-nucleotide polymorphism, phylogenetic analysis

## Abstract

African swine fever virus (ASFV), classified as genotype II, was introduced into Georgia in 2007, and from there, it spread quickly and extensively across the Caucasus to Russia, Europe and Asia. The molecular epidemiology and evolution of these isolates are predominantly investigated by means of phylogenetic analysis based on complete genome sequences. Since this is a costly and time-consuming endeavor, short genomic regions containing informative polymorphisms are pursued and utilized instead. In this study, sequences of the central variable region (CVR) located within the B602L gene were determined for 55 ASFV isolates submitted from 526 active African swine fever (ASF) outbreaks occurring in 23 different regions across the Russian Federation (RF) between 2013 and 2017. The new sequences were compared to previously published data available from Genbank, representing isolates from Europe and Asia. The sequences clustered into six distinct groups. Isolates from Estonia clustered into groups 3 and 4, whilst sequences from the RF were divided into the remaining four groups. Two of these groups (5 and 6) exclusively contained isolates from the RF, while group 2 included isolates from Russia as well as Chechnya, Georgia, Armenia, Azerbaijan and Ukraine. In contrast, group 1 was the largest, containing sequences from the RF, Europe and Asia, and was represented by the sequence from the first isolate in Georgia in 2007. Based on these results, it is recommended that the CVR sequences contain significant informative polymorphisms to be used as a marker for investigating the epidemiology and spread of genotype II ASFVs circulating in the RF, Europe and Asia.

## 1. Introduction

African swine fever (ASF) is a fatal viral disease of domestic pigs and wild boars of all ages. The causative agent is the only known double-stranded DNA arbovirus, ASF virus (ASFV), which belongs to the *Asfarviridae* family in the genus Asfivirus. The genome ranges from 170 to 190 kilobase pairs (kbp) and encodes more than 150 open reading frames (ORFs), depending on the viral strain [1,2]. Traditionally, the disease has been confined to sub-Saharan Africa and the Italian island of Sardinia, but sporadic epidemics have affected a number of countries throughout the 20th century. However, in 2007, ASFV was first reported in Georgia, and from there, it spread to Armenia and Azerbaijan and subsequently into the Russian Federation (RF), Ukraine and Belarus [3]. In 2014, four European Union countries, Lithuania, Poland, Latvia, and Estonia, reported ASF prior to its subsequent spread to Belgium, Bulgaria, Czech Republic, Hungary, Romania and Slovakia [4]. In 2018, the disease spread to China, the world’s largest pig-producing country, resulting in devastating economic losses to the country [5]. In recent years, ASF has rapidly spread beyond China to neighboring countries, including Mongolia, Cambodia, North Korea, South Korea, the Philippines and India [6]. In July 2021, ASFV genotype II was reported in the Dominican Republic and Haiti, and additionally in Germany, Greece and Italy [7].

Currently, ASFV is classified into 24 genotypes based on sequence data from the C-terminal region of the open reading frame (ORF) B646L, encoding the major capsid protein p72 [8,9]. This gene region is frequently used to investigate the molecular epidemiology of ASF by determining and comparing the genotypes circulating in a region [9]. The additional differentiation of closely related viruses into sub-groups has been subsequently performed using the p54 locus (E183L), the central variable region (CVR) in the B602L gene [10], tandem repeat sequence (TRS) insertion in the intergenic regions (IGR) and multigene family (MGF) 505 9R/10R [11]. Additional genome markers, K145R, MGF 505-5R and O174L, have been used to differentiate isolates from Poland [12]. A novel 14 base pair (bp) TRS insertion of CAGTAGTGATTTTT was identified in the O174L gene of certain isolates from Poland [12]. Complete genome sequences from isolates in the RF recommended that MGF-360-10L, MGF-505-9R and I267L be included as additional genome markers, since they have the resolving capabilities of separating isolates from the RF, EU and China geographically into an eastern and western cluster [13]. The CVR region is frequently targeted for sequence analyses due to unique mutations capable of resolving phylogenies at a regional level, including clustering isolates into groups and sub-types [9,14]. Isolates from Africa were recently divided into various sub-types, based on informative polymorphisms observed within this gene region [15]. Despite the large number of ASFV genotype II outbreaks in Europe and Asia, variants in the CVR have only been described for isolates from Estonia, classifying these isolates into three uniquely distinctive CVR variant groups [16]. Based on the resolving power of this gene region in isolates from Africa and the polymorphisms observed in isolates from Estonia, it was hypothesized that this gene region could be used as a fast and cost-effective method to investigate the epidemiology, evolution and molecular relatedness of large numbers of isolates from the RF. The aim of this study was to characterize the CVR sequences of 55 ASFV isolates, each representing closely linked outbreaks from 23 different regions of the RF during 2013–2017, and subsequently determine the phylogenetic relationship between these isolates with ASFVs from Europe and Asia.

## 2. Materials and Methods

### 2.1. Ethics Statement

No animals were used during this study, but samples from clinically infected domestic pigs and wild boars were submitted for the laboratory confirmation of ASFV to the national reference laboratory at the Federal Center for Animal Health (FGBI “ARRIAH”) in Vladimir, Russia.

### 2.2. Isolates and Virus Identification

In this study, 55 ASFV PCR-positive samples were selected as representatives of the 526 outbreaks reported in 23 different regions of the RF at different times during the period 2013–2017. A brief summary of these isolates is provided in Table 1.

Blood or organ tissue samples were collected from domestic pigs (DPs) and either hunted or dead wild boars (WBs). These samples were refrigerated and shipped to FGBI ARRIAH within 24 h of collection. Viral DNA was extracted using the DNeasy Blood & Tissue Kit (Qiagen, Germany) following the manufacturer’s recommendations, and the presence of ASFV nucleic acids was determined via real-time PCR according to recommendations of the OIE [17]. 

### 2.3. Sequence Alignment and Phylogenetic Analysis

A 233bp region of the CVR (B602L) gene was PCR-amplified as previously described using the primer pairs ORF9L-F (5′-AATGCGCTCAGGATCTGTTAAATCGG-3′) and ORF9L-R (5′-TCTTCATGCTCAAAGTGCGTATACCT-3′) [10,18]. These amplicons were submitted for Sanger sequencing at the FGBI “ARRIAH” institute using both primers incorporated during the generation of the amplicons. Both sequences were assembled to generate a consensus sequence representing the CVR gene of each isolate. Nucleotide sequences were aligned and compared to corresponding sequences from Genbank (Supplementary Table S1) using Bioedit v7.2.5 software (by Hall, T.A., CA, USA). The phylogenetic relatedness of these sequences was analyzed using Maximum Likelihood under General Time Reversal (GTR + GI = 4), with the consideration of all the sites to account for the gaps due to deletions in the analysis. The sequence of ASFV genotype I Liv13/33 was included as an outlier.

## 3. Results

**Figure 1:**
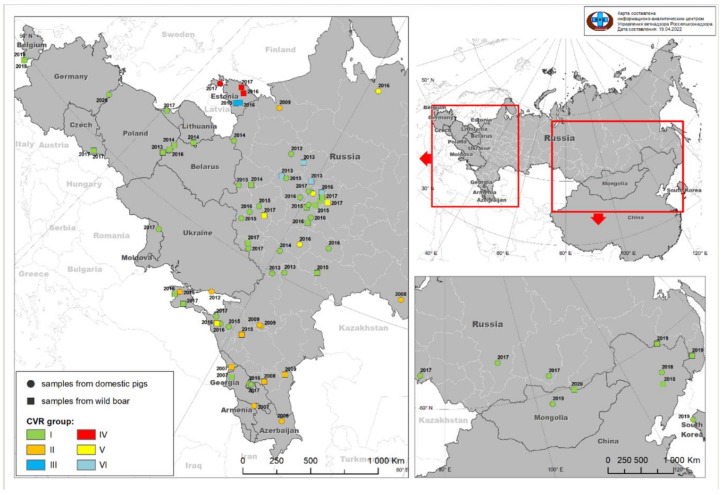
Distribution of the ASFVs from the territory of the RF, EU and Asia based on the sub-division of sequences using the CVR locus. Sequences represent ASFV samples obtained between 2007 and 2020, from either DPs (circles) or WBs (squares). The sub-division of CVR sequences into six groups is graphically presented as indicated by the key provided in the figure.

**Figure 2:**
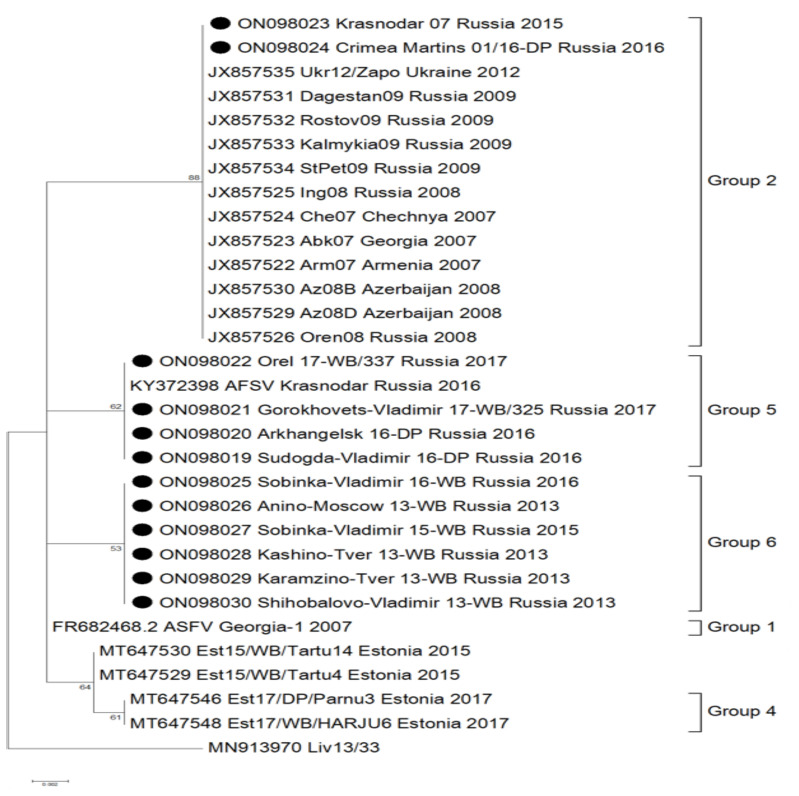
Maximum-likelihood phylogenetic tree based on the 281 bp partial sequence of the B602L gene (CVR) of the ASFV genome. Included in this analysis are the 55 sequences generated in this study from isolates within the RF, as well as sequences obtained from Genbank. Solid black circles are used to identify isolates from this study that belonged to groups other than group 1 (Georgia 2007/1); isolates that showed 100% identity to Georgia 2007/1 are not shown.

The partial gene region of ORF B602L from 55 ASFV isolates, representing 526 outbreaks from 23 different regions within the RF during 2013–2017, were amplified and Sanger sequenced. The sequences were compared to data available from GenBank, representing additional genotype II ASFVs obtained from Europe and Asia. These sequences were aligned, and single-nucleotide polymorphisms (SNPs) were described using isolate Georgia-2007/1 (FR682468.2) as a reference. By comparing the new sequences with all the available data from Europe and Asia, the sequences were sub-divided into six distinct groups. These sub-divisions were based on five SNPs and one deletion and are subsequently described in detail (Figure 1, Figure 2 and Figure S1). 

The first group (1) consisted of isolates that shared 100% sequence identity to Georgia 2007/1 (FR682468.2). This group included the majority of the new and old isolates from the RF as well as isolates from Europe and Asia with the exception of Estonia (Table 1, Supplementary Figure S1).

Sequences belonging to group 2 were characterized by two unique SNPs in contrast to the reference sequence Georgia 2007/1 (FR682468.2). A synonymous (C/T) SNP at position 480 and a non-synonymous (A/G) SNP at position 616 were described within these sequences (Supplementary Figure S1). The latter SNP resulted in threonine (T) exchange of an alanine (A) at position 206 of the complete B602L predicted protein (Supplementary Figure S2). This group contained 14 isolates from Eastern Europe, submitted between 2007 and 2014, with 12 sequences obtained from GenBank [19]. The sequences represent samples from Chechnya in 2007 (Che07; JX857524); Georgia in 2007 (Abk07; JX857523); Armenia in 2007 (Arm 07; JX857522); Azerbaijan in 2008 (Az08B; JX857530 and Az08D; JX857529); Ukraine in 2012 (Ukr12/Zapo; JX857535); and the RF between 2008 to 2016 (Oren08; JX857526, Ing08; JX857525, StPet09; JX857534; Kalmykia09; JX857533, Rostov 09; JX857532, Dagestan09; JX857531 as well as from this study: Krasnodar 07/15; ON098023 and Crimea 01\6 Martins; ON098024). Isolates from the RF belonging to this group were mainly submitted from the south-western regions of the RF (Figure 1).

Groups 3 and 4 were previously identified in a study performed by Vilem et al., 2020. Group 3 was characterized by a 35 bp deletion at position 481, resulting in an amino acid deletion of CASMCADTNVDT (Supplementary Figures S1 and S2). Group 4 contained a non-synonymous (A/G) SNP at position 506, which resulted in a cysteine (C) to tyrosine (Y) exchange at the predicted amino acid position 193 of the complete B602L protein. All sequences analyzed in both of these groups were obtained from Genbank, representing isolates unique to Estonia submitted between 2014 and 2017 [16] (Figure 2).

Group 5 had a non-synonymous (A/G) mutation at position 601, resulting in a lysine (K) exchange of glutamic acid (E) at the predicted amino acid position 201 of the B602L protein (Supplementary Figures S1 and S2). This SNP was observed in six isolates selected and sequenced in this study, representing outbreaks from the RF submitted to the laboratory in 2016 and 2017 (Sudogda-Vladimir 16-DP, Arkhangelsk 16-DP, Tambov 2016, Krasnodar 2016, Gorokhovets-Vladimir 17-WB/325 and Orel 17-WB/337) (Figure 2).

Lastly, group 6 was characterized by a single (A/T) SNP at position 459, identified in six isolates selected and analyzed in this study, representing samples submitted between 2013 and 2016 in Russia (Anino-Moscow 13-WB, Kashino-Tver 13-WB, Karamzino-Tver 13-WB, Shihobalovo-Vladimir 13-WB, Sobinka-Vladimir 15-WB and Sobinka-Vladimir 16-WB) (Figure 2). This is a synonymous SNP involving leucine (L) at amino acid position 153 (Supplementary Figures S1 and S2).

As represented by the phylogenetic tree, all isolates belonging to genotype II were clustered into six different groups, based on the mutations identified in the CVR of the virus genome. Group 1 constituted the largest number of isolates and shared 100% identity to the Georgia 2007/1 sequence. Isolates from the RF could be divided into four distinct groups: 1, 2, 5 and 6. Isolates from Azerbaijan, Georgia, Armenia and Ukraine clustered in group 2 along with samples from the RF (Figure 2). The latter had samples that were unique to groups 5 and 6 (Figure 2). Isolates from Estonia were sub-divided into groups 3 and 4, resulting in both groups being unique to this country (Figure 2). 

Three of the five SNPs utilized during the demarcation of the six groups were non-synonymous, resulting in T206A exchange in 16 isolates and E201K exchange in 7 isolates submitted from the RF (Supplementary Figure S2). In addition, C169Y exchange was uniquely described for 24 isolates from Estonia in 2017 (Supplementary Figure S2) [16]. The isolates from Estonia in 2015 and 2016 were the only sequences that had a deletion of one of the tetramer-tandem repeats (CADT, NVDT and CASM) containing only seven of the eight tetramers (Supplementary Figure S2). The synonymous SNP in group six reduced the number of groups based on amino acid differentiation to five, compared to the six groups described based on nucleotide analysis (Supplementary Figures S1 and S2).

## 4. Discussion

Since the introduction of ASF into Georgia in 2007, the disease has been spreading in an unprecedented manner across Eurasia. Fear of ASF emergence in the territory, either in domestic pigs or in wild boar populations, exists in many countries currently still free from the disease [20]. From 2007 to 04.04.2022, about 62,351 outbreaks/cases of ASF have been reported in the territory of the RF, Europe, Asia and the Caribbean [7]. Of these, 2139 outbreaks were reported in the RF. Studies on the epidemiology of ASFVs in Europe indicated that wild boars and the products of affected pigs were the largest contributing factors pertaining to the spread of the disease in this region [21]. The subsequent monitoring of outbreaks and tracking of virus movements using genetic tools are therefore imperative in an efficient ASF control strategy. The gold standard in unraveling the relationship between ASFVs is based on the elucidation of a complete genome sequence of individual isolates and subsequent comparative analysis involving multiple ASFVs [22]. However, this procedure is time-consuming, labor-intensive and expensive [23]. Single genomic loci could provide a fast and cost-effective medium to resolve ASFV isolates based on differences in informative SNPs and size variations. Potential loci that could be used to resolve the molecular epidemiology of closely related genotype II ASFVs in Russia, Europe and Asia include I267L, MGF 505-5R and K145R and the CVR locus (B602L) [10,12,13,19]. Based on the analysis of O174L, three variants of ASFV were identified in Poland, whilst a single SNP in the K145R gene identified two additional variants of the virus [12]. Additionally, the IGR (I73R/I329L) verified the circulation of three variants [12]. Isolates in Vietnam clustered in a single group identical to Georgia 2007 based on the sequence analysis of the CVR, while the same isolates were divided into three groups based on their IGR sequences [6]. The CVR gene region is frequently applied to the intra-genotype differentiation of ASFVs circulating in African countries [14,15].

In this study, the partial B602L gene containing the CVR locus of 55 ASFVs, selected to represent outbreaks from different regions of the RF, was determined and compared to previously published sequence data from Europe and Asia. Based on these sequence analyses, samples from the RF were sub-divided into four unique clusters. In addition, ASFVs from Europe and Asia were divided into six distinct groups based on the same region (Supplementary Figure S1) [16]. This is due to the unique sequences previously described in Estonia between 2015 and 2017 [16]. 

The data generated in this study clustered the isolates into four groups that mirror the spatial or temporal origins of the isolates from the RF (Figure 1). This suggests that the outbreaks were highly clonal and that this marker could be used to track the origin and spread of viruses in future epidemiological studies. The identification of genetically highly related strains observed over multiple years within the same geographical location is indicative of the localized circulation of ASFV in possibly wild boar populations, which has been suggested by previous studies [24].

Interestingly, there were four exceptions to the observed spatiotemporal clustering of the defined groups. Two isolates from group 2, St. Petersburg in 2009 and Orenburg in 2008, as well as two isolates in group 5, Arkhangelsk in 2016 and Krasnodar in 2016, were submitted between 1300 and 1500 km from the nearest isolate belonging to the same cluster (Figure 1). This vast distances between outbreaks suggest possible transboundary incursions from outside the area of study, possibly related to movement of domestic pigs or pig-based products, rather than the localized wild boar transmission pathway. Additionally, group 2 included isolates from Armenia, Georgia, Azerbaijan and Ukraine, which further supports the hypothesis of the possible involvement of human actions in the spread of the disease across international borders [19]. 

## 5. Conclusions

This is the first study to differentiate isolates from the territory of the RF, based on CVR sequences. The sequences were clustered into four groups that mirrored the spatial and/or temporal distribution of the outbreaks represented by these isolates from the RF. Based on these results, representatives of outbreaks submitted from the same regions between 2018 and 2022 will be analyzed based on the CVR. The aim of these studies will be to identify any novel mutations and to evaluate if the variants identified within this study are still circulating in the same regions or have spread to new regions.

## Figures and Tables

**Table 1:** Brief characteristics of samples used in this study, including the collection year and location as well as the GenBank accession number and the group each sequence is assigned to.

No.	Collection Date	Host	Isolate Name	Region	Accession Number of the CVR Locus	Group
Seq1	2016	DP	Sudogda-Vladimir_16-DP	Vladimir	ON098019	5
Seq2	2016	DP	Arkhangelsk_16-DP	Arkhangelsk	ON098020	5
Seq3	2017	WB	Gorokhovets-Vladimir_17-WB/325	Vladimir	ON098021	5
Seq4	2017	WB	Orel_17-WB/337	Orel	ON098022	5
Seq5	2015	DP	Krasnodar_07/15	Krasnodar	ON098023	2
Seq6	2016	DP	Crimea_Martins_01/16-DP	Crimea	ON098024	2
Seq7	2016	WB	Sobinka-Vladimir_16-WB	Vladimir	ON098025	6
Seq8	2013	WB	Anino-Moscow_13-WB	Moscow	ON098026	6
Seq9	2015	WB	Sobinka-Vladimir_15-WB	Vladimir	ON098027	6
Seq10	2013	WB	Kashino-Tver_13-WB	Tver	ON098028	6
Seq11	2013	WB	Karamzino-Tver_13-WB	Tver	ON098029	6
Seq12	2013	WB	Shihobalovo-Vladimir_13-WB	Vladimir	ON098030	6
Seq13	2013	DP	Boguchary-Voronezh_13-DP/2051	Voronezh	ON098031	1
Seq14	2013	DP	Volgograd_13-DP/2078	Volgograd	ON098032	1
Seq15	2013	DP	Volgograd_13-DP/2059	Volgograd	ON098033	1
Seq16	2014	DP	Voronezh_Agro_14-DP	Voronezh	ON098034	1
Seq17	2014	WB	Grafskoe-Belgorod_14-WB	Belgorod	ON098035	1
Seq18	2014	WB	Odintsovo_02/14-Moscow_14-WB	Moscow	ON098036	1
Seq19	2014	WB	Gribovo-Kaluga_14-WB	Kaluga	ON098037	1
Seq20	2014	WB	Vasilenki-Kaluga_08/14-WB	Kaluga	ON098038	1
Seq21	2014	DP	Antonovo-Pskov_14-DP	Pskov	ON098039	1
Seq22	2015	WB	Ryazan_15-WB	Ryazan	ON098040	1
Seq23	2015	DP	Saratov_01/15	Saratov	ON098041	1
Seq24	2015	DP	Guskhrustalny-Vladimir_15-DP	Vladimir	ON098042	1
Seq25	2015	WB	Ryazan(Autumn)_15-WB	Ryazan	ON098043	1
Seq26	2015	DP	Bolhovsky-Orel_15-DP	Orel	ON098044	1
Seq27	2015	DP	Kurtnikovo-Moscow_15-DP	Moscow	ON098045	1
Seq28	2015	WB	Sashino-Vladimir_15-WB	Vladimir	ON098046	1
Seq29	2015	DP	Smolensk_15-DP	Smolensk	ON098047	1
Seq30	2015	DP	Kursk_15-DP	Kursk	ON098048	1
Seq31	2015	DP	Sokolckie_Vol-Krasnodar_15-DP	Krasnodar	ON098049	1
Seq32	2015	DP	Orel_15-DP	Orel	ON098050	1
Seq33	2017	DP	Krasnodar_07/17	Krasnodar	ON098051	1
Seq34	2016	DP	South_Osetia_16/DP/2325	South Ossetia	ON098052	1
Seq35	2016	WB	Ryazan_03/16	Ryazan	ON098053	1
Seq36	2016	WB	Ryazan_07/16-WB	Ryazan	ON098054	1
Seq37	2016	DP	Shatsky_-_Ryazan_16-DP	Ryazan	ON098055	1
Seq38	2016	DP	Orel-Mtsensk_16-DP	Orel	ON098056	1
Seq39	2016	DP	Kropotkin-Krasnodar_16-DP	Krasnodar	ON098057	1
Seq40	2016	WB	Crimea_16-WB	Crimea	ON098058	1
Seq41	2016	DP	Vrachovo-Moscow_16-DP	Moscow	ON098059	1
Seq42	2016	DP	Penza_16-DP	Penza	ON098060	1
Seq43	2017	DP	Kolchugino-Vladimir_17-DP/5662	Vladimir	ON098061	1
Seq44	2017	DP	Sobinka-Vladimir_17-DP/5660	Vladimir	ON098062	1
Seq45	2017	DP	Sobinka-_Vladimir_17-DP/328	Vladimir	ON098063	1
Seq46	2017	DP	Omsk_17-DP/5665	Omsk	ON098064	1
Seq47	2017	WB	Vyaznikovski-Vladimir_17-WB/330	Vladimir	ON098065	1
Seq48	2017	DP	Irkutsk_17-DP/447	Irkutsk	ON098066	1
Seq49	2017	DP	South_Osetia_17-DP/2196	South Osetia	ON098067	1
Seq50	2017	WB	Crimea_17-WB/470	Crimea	ON098068	1
Seq51	2017	DP	Krasnoyarsk_10/2017	Krasnoyarsk	ON098069	1
Seq52	2017	DP	Omsk_10/2017	Omsk	ON098070	1
Seq53	2017	DP	Belgorod_10/17	Belgorod	ON098071	1
Seq54	2017	WB	Belgorod_12/17	Belgorod	ON098072	1
Seq55	2017	DP	Kaliningrad_12/17	Kaliningrad	ON098073	1

DP = domestic pig; WB = wild boar.

## Data Availability

The datasets presented in this study were submitted to the Genbank database (ON098019-ON098073). The names of the isolates and accession number(s) can be found in the article/Table 1.

## Supplementary Materials

The following supporting information can be downloaded at: www.mdpi.com/article/10.3390/pathogens11080919/s1, Figure S1: Alignment of CVR (B602L) nucleotide sequences of isolates representing the six groups; Figure S2: Alignment of the predicted partial amino acidic sequence of pB602L from representative isolates of each of six groups; Table S1: List of isolates obtained from Genbank used in this study to compare with new isolates from the RF.

## Abbreviations

ASFV: African swine fever virus; PCR, polymerase chain reaction; CVR, central variable region; kbp, kilobase pair; IGR, intergenic region; TRS, tandem repeat sequence; UPGMA, unweighted pair group method with arithmetic mean.
