# Supplementary material for: Genetic Characterization of the Central Variable Region in African Swine Fever Virus Isolates in the Russian Federation from 2013 to 2017
Source: Pathogens. 2022 Aug 15;11(8):919. doi: 10.3390/pathogens11080919 (PMC9413668; doi:10.3390/pathogens11080919)
Supplement: Supplementary file 1 [file pathogens-11-00919-s001.zip › Supplementary Table S1.pdf]

Supplementary Table S1. List of isolates obtained from Genbank used in this study to compare with new isolates from the RF.

Isolate	Country	Accession number	CVR Group
Georgia_2007/1	Georgia	FR682468.2	1
Abk07	Georgia	JX857523.1	2
Arm07	Armenia	JX857522.1	1
Az08D	Azerbaijan	JX857529.1	2
Az08B	Azerbaijan	JX857530.1	2
Ukr12/Zapo	Ukraine	JX857535.1	2
Krasnodar2016	Russia	KY372398.1	5
ASFV/Ulyanovsk_19/WB-5699	Russia	MW306192.1	5
Tambov2016	Russia	KY372399.1	5
Che07	Russia	JX857524.1	2
Ing08	Russia	JX857525.1	2
Oren08	Russia	JX857526.1	2
NO08/Av	Russia	JX857527.1	1
NO08/Ap	Russia	JX857528.1	1
Rostov09	Russia	JX857532.1	2
Dagestan09	Russia	JX857531.1	2
Kalmykia09	Russia	JX857533.1	2
StPet09	Russia	JX857534.1	2
Tver1112/Zavi	Russia	KJ627202.1	1
Tver0312/Novo	Russia	KJ627201.1	1
Tver0312/Torjo	Russia	KJ627200.1	1
Tver0712/Les	Russia	KJ627199.1	1

Tver0812/Bolo	Russia	KJ627198.1	1
Tver0511/Torjo	Russia	KJ627197.1	1
ASFV/Kaliningrad_18/WB-9734	Russia	OM966721	1
ASFV/Kaliningrad_18/WB-9735	Russia	OM966716	1
ASFV/Kaliningrad_18/WB-9763	Russia	OM966717	1
ASFV/Kaliningrad_18/WB-12524	Russia	OM966715	1
ASFV/Kaliningrad_18/WB-9766	Russia	OM966718	1
ASFV/Kaliningrad_18/WB-12523	Russia	OM966714	1
ASFV/Kaliningrad_18/WB-12516	Russia	OM966720	1
ASFV/Kaliningrad_19/WB-10168	Russia	OM966719	1
ASFV/Kabardino-Balkaria_19/WB-964	Russia	MT459800.1	1
ASFV/Odintsovo_2014/WB	Russia	KP843857.1	1
ASFV/Primorsky_19/WB-6723	Russia	MW306191.1	1
ASFV/Amur_19/WB-6905	Russia	MW306190.1	1
ASFV/Zabaykali_2020/WB-5314	Russia	MZ325862.1	1
Amur/19.08.19	Russia	MT840357.1	1
Irkutsk2017	Russia	KY938010.1	1
Kursk_2015	Russia	KY367260.1	1
Orel2015	Russia	KY372397.1	1
ASFV/Kaliningrad_17/WB-	Russia	OM799941	1

13869			
Est16/WB/Tartu17	Estonia	MT647531.1	3
Est15/WB/Tartu14	Estonia	MT647530.1	3
Est15/WB/Tartu4	Estonia	MT647529.1	3
Est15/WB/Tartu2	Estonia	MT647528.1	3
Est15/WB/Tartu1	Estonia	MT647527.1	1
Est15/WB-Tartu37	Estonia	MT647534.1	1
Est15/WB-Tartu39	Estonia	MT647535.1	1
Est16/WB/Tartu19	Estonia	MT647533.1	3
Est16/WB/Tartu18	Estonia	MT647532.1	3
Est15/WB-Tartu40	Estonia	MT647540.1	1
Est16/WB-Tartu56	Estonia	MT647543.1	1
Est15/WB-Tartu47	Estonia	MT647542.1	1
Est17/DP/Parnu1	Estonia	MT647544.1	1
Est17/DP/Parnu2	Estonia	MT647545.1	1
Est17/DP/Parnu3	Estonia	MT647546.1	4
Est17/DP/Parnu4	Estonia	MT647547.1	4
Est17/WB/HARJU6	Estonia	MT647548.1	4
Est17/WB/LANE12	Estonia	MT647549.1	1
Est17/WB/Parnu11	Estonia	MT647550.1	1
Est17/WB/Parnu12	Estonia	MT647551.1	1
Est17/WB/Parnu13	Estonia	MT647552.1	1
Est17/WB/Parnu14	Estonia	MT647553.1	1
Est16/WB/LANE22	Estonia	MT647554.1	1
Est16/WB/LANE23	Estonia	MT647555.1	1
Est17/WB/Parnu15	Estonia	MT647556.1	1

Est17/WB/LANE27	Estonia	MT647557.1	1
Est17/WB/Parnu16	Estonia	MT647558.1	1
Est17/WB/Harju12	Estonia	MT647559.1	1
Est17/WB/Parnu17	Estonia	MT647560.1	1
Est17/WB/Parnu18	Estonia	MT647561.1	1
Est17/WB/Parnu19	Estonia	MT647562.1	1
Est17/WB/Parnu20	Estonia	MT647564.1	1
Est17/WB/Harju13	Estonia	MT647563.1	1
Est17/WB/LANE31	Estonia	MT647565.1	1
Est17/DP/Parnu5	Estonia	MT647566.1	1
Est17/DP/Parnu6	Estonia	MT647567.1	1
ASFV_POL/2015/Podlaskie	Poland	MH681419.1	1
ASFV_Pol16_20538_09	Poland	MG939584.1	1
ASFV_Pol16_29413_023	Poland	MG939586.1	1
ASFV_Pol16_20540_010	Poland	MG939585.1	1
ASFV_Pol16_20186_07	Poland	MG939583.1	1
ASFV_Pol17_55892_C754	Poland	MT847620.1	1
ASFV_Pol17_31177_O81	Poland	MT847622.1	1
ASFV_Pol17_04461_C210	Poland	MG939588.1	1
ASFV_Pol17_03029_C201	Poland	MG939587.1	1
ASFV_Pol17_05838_C220	Poland	MG939589.1	1
ASFV_Pol18_28298_O111	Poland	MT847621.1	1
ASFV_Pol19_53050_C1959/19	Poland	MT847623.2	1
Pol14/Sz	Poland	KJ627206.1	1
Pol14/Krus_pB602L	Poland	KJ627207.1	1
RO/SM/2017/CVR	Romania	MN809122.1	1

LT14/1482	Lithuania	KJ627205.1	1
LT14/1490	Lithuania	KJ627204.1	1
Bel13/Grodno	Belarus	KJ627203.1	1
ASFV/LT14/1490	Lithuania	MK628478.1	1
ASFV_CzechRepublic_2017/1	CzechRepublic	LR722600.1	1
ASFV_Moldova_2017/1	Moldova	LR722599.1	1
ASFV_Belgium_2018/1	Belgium	LR536725.1	1
ASFV_Germany_2020/1	Germany	LR899193.1	1
ASFV/pig/China/CAS19-01/2019	China	MN172368.1	1
ASFV_China_DB/LN/2018	China	MK333181.1	1
ASFV_China_ASFV-wbBS01	China	MK645909.1	1
ASFV_China/2018/AnhuiXCGQ	China	MK128995.1	1
ASFV_China_Pig/HLJ/2018	China	MK333180.1	1
ASFV_Wuhan_2019-1	China	MN393476.1	1
ASFV_CN/2019/InnerMongolia-AES01	China	MK940252.1	1
ASFV-wbBS01	China	MK238344.1	1
IND/AS/SD-02/2020	India	MT642590.1	1
IND/AS/SD-13/2020	India	MT642591.1	1
IND/AR/SD-61/2020	India	MT642593.1	1
IND/AR/SD-59/2020	India	MT642592.1	1
VNUA_NA-ASF6	VietNam	MZ812718.1	1
ASF/POB(BLOOD)/VietNam/2019	VietNam	MW451101.1	1
ASF/POT(TISSUE)/VietNam/2	VietNam	MW451100.1	1

019			
ASF/P1/VietNam/2019	VietNam	MW451102.1	1
ASF/P5/VietNam/2019	VietNam	MW451103.1	1
ASF/P10/VietNam/2020	VietNam	MW451104.1	1
ASF/P15/VietNam/2020	VietNam	MW451105.1	1
VNUA_Hanoi-ASF9	Vietnam	MZ812719.1	1
Korea/Pig/Paju1/2019	Southern-Korea	MN631140.1	1
