# Supplementary figures and images for: Genetic Characterization of the Central Variable Region in African Swine Fever Virus Isolates in the Russian Federation from 2013 to 2017
Source: Pathogens. 2022 Aug 15;11(8):919. doi: 10.3390/pathogens11080919 (PMC9413668; doi:10.3390/pathogens11080919)

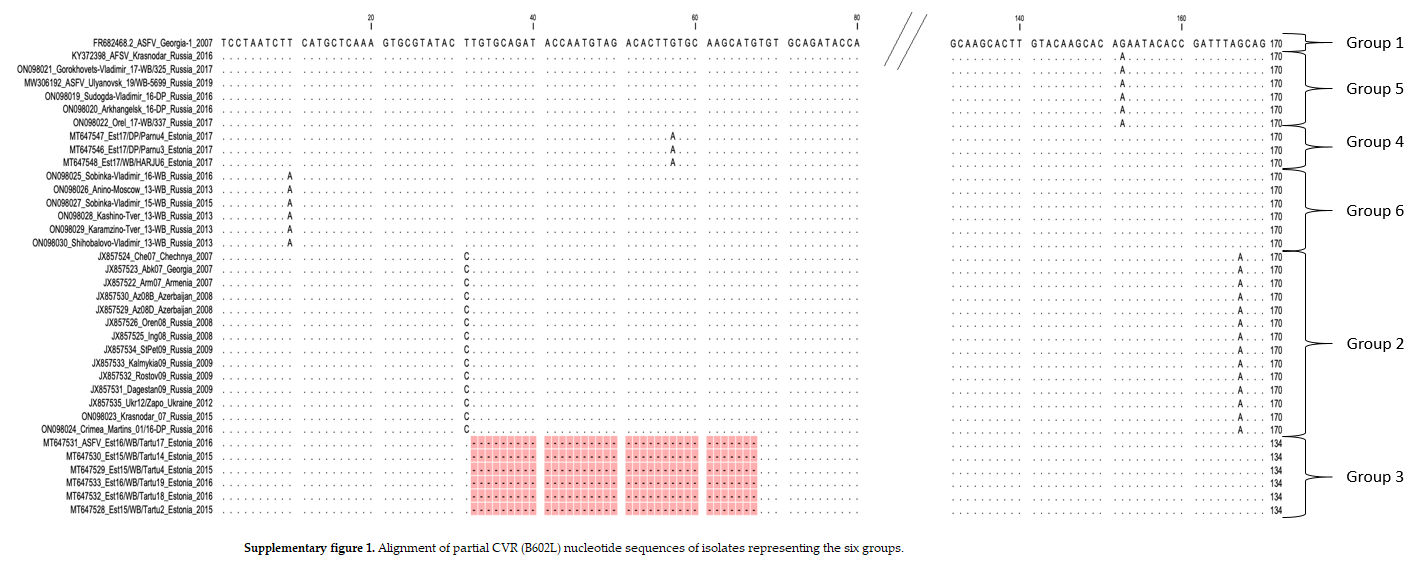

Supplement: Supplementary file 1 [file pathogens-11-00919-s001.zip › Supplementary Figure S1.tif]

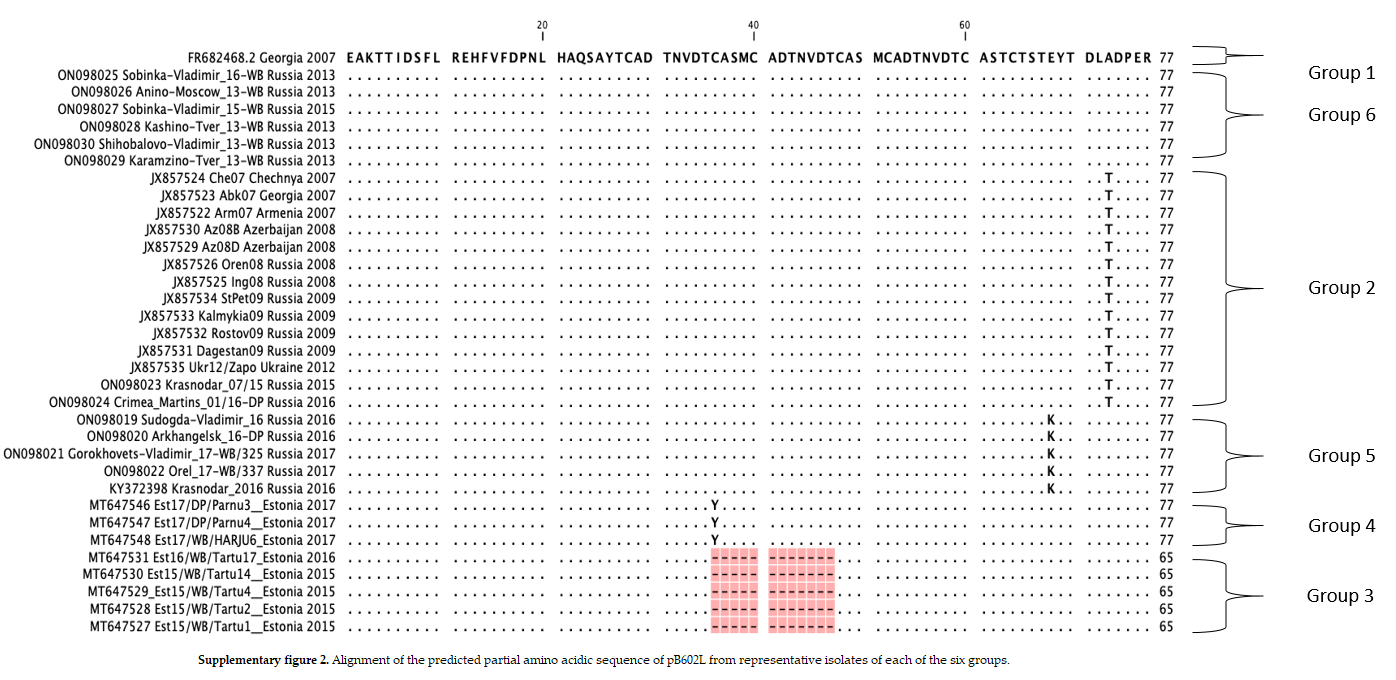

Supplement: Supplementary file 1 [file pathogens-11-00919-s001.zip › Supplementary Figure S2.tif]
